# Forgiveness in the Modulation of Responsibility in a Sample of Italian Adolescents with a Tendency towards Conduct or Obsessive–Compulsive Problems

**DOI:** 10.3390/brainsci11101333

**Published:** 2021-10-09

**Authors:** Carlo Buonanno, Enrico Iuliano, Giuseppe Grossi, Francesco Mancini, Emiliana Stendardo, Fabrizia Tudisco, Barbara Pizzini

**Affiliations:** 1 School of Cognitive Psychotherapy srl, 00162 Roma, Italy; iulianoenrico@libero.it (E.I.); g.grossi@hotmail.it (G.G.); mancini@apc.it (F.M.); stendardoe@gmail.com (E.S.); fabrizia-tudisco@virgilio.it (F.T.); 2 InMovement Center, 04022 Fondi, Italy; 3 Department of Human Sciences, University of Studies Guglielmo Marconi, 00193 Roma, Italy; 4 Department of Psychology, University of Campania “Luigi Vanvitelli”, 81100 Caserta, Italy

**Keywords:** forgiveness, responsibility, guilt, obsessive-compulsive problems, conduct problems, adolescence

## Abstract

Although obsessive–compulsive disorder (OCD) and the conduct disorders (CD) express a contrasting symptomatology, they could represent different answers to a common matrix about morality. In the literature, some theoretical models describe people with OCD as individuals who experience high levels of responsibility and guilt. On the other hand, adolescents with a CD are described as if they do not feel guilty at all or consider anti-social purposes as more important than existing moral purposes. The aims of this study were to investigate the role of forgiveness in responsibility and guilt levels and to test whether this putative relation was influenced by tendencies towards obsessive–compulsive problems (OCP) or conduct problems (CP). In total, 231 adolescents aged between 16 and 18 years were self-assessed using a Youth Self-Report, Child Responsibility Attitudes Questionnaire, Heartland Forgiveness Scale, and Test Of Self-Conscious Affect. The results show that self-forgiveness predicted responsibility levels, while guilt was predicted by self-forgiveness and situation-forgiveness. Moreover, mediation analyses revealed that the effects of OCP on responsibility and guilt were mediated by self-forgiveness and situation-forgiveness. Regarding CP, no mediated effects were found. In conclusion, lower proneness to forgive increases responsibility and guilt, and this is particularly evident in subjects with higher levels of OCP.

## 1. Introduction

Morality can be defined as a set of prescriptive norms concerning others’ welfare, rights, fairness, and justice [1,2]. It refers to the way people choose to live their lives according to a set of principles that lead their decisions about what is right or wrong, good or evil. Theoretical constructs of morality can be considered as consisting of three main dimensions: a pure cognitive aspect regarding moral reasoning (e.g., a specific type of reasoning, by which moral principles provide the grounds for moral judgments) [3]; moral behaviours (e.g., expression of behaviours that benefit society) [4]; and moral emotions (e.g., shame, guilt, sympathy, and empathy) [5].

Seeds of morality emerge early in children with typical trajectories of development. Young children (i.e., 2–3 years old) spontaneously engage in prosocial behaviours and, conversely, want to avoid antisocial behaviour [6,7]. Regarding moral reasoning, from preschool age onwards, children show an ability to recognise and protest injustice or harmful behaviour [8,9,10,11], suggesting that children grasp concepts of harm and unfairness. Regarding moral emotions, Frick et al. [12] argued that, while in early childhood it may be difficult to distinguish between moral emotions, they become more differentiated over development, with guilt becoming more specifically related to prosocial behaviour [13]. Therefore, as children become adolescents, they able to integrate cognitive, emotional, and behavioural aspects of their morality by applying their moral judgments and emotions to social situations while they coordinate competing concerns in different contexts [14]. 

Among moral emotions, guilt is the one more strongly associated with morality (especially with prosocial behaviours). It is believed that it influences human behaviour, particularly when transgressions that violate social prescriptions happen. Indeed, guilt has been defined as thoughts and feelings of distress associated with transgressions [15,16,17]. Moreover, in the field of social psychology, guilt is considered an adaptive emotion able to improve social relationships through the development of an empathic concern for the well-being of others [18,19,20]. 

Always adopting an interpersonal view of guilt, some authors [21] relate the moral emotion of guilt with the perceived sense of personal responsibility by sustaining that guilt is a moral emotion composed of five factors (including distress and beliefs about responsibility) interacting with each other. In this view, the magnitude of guilt is proportional to the relative importance of each factor, namely, to perceived responsibility. 

Responsibility can be described as the belief that a person possesses a pivotal power to provoke or prevent crucial negative consequences of their behaviour [22]. Results have shown that a perceived sense of responsibility is mediated by guilt, even if the contrary is not true [23]. This result highlights that those differences in guilt, although strongly influenced by responsibility, cannot be accounted for entirely in terms of responsibility [24,25].

Therefore, considering all the above, adolescents are called upon to find the right balance between social and personal demands on moral issues. Obstacles to this balance can lead to symptom spectra, such as obsessive–compulsive disorder on one hand or conduct disorders on the other.

In obsessive–compulsive disorder (OCD), a strong sensitivity to guilt is considered a crucial element, particularly in cognitive-behavioural models [22,25,26,27,28]. Excessive responsibility (also called ‘inflated responsibility’) has been suggested as a central cognitive variable associated with this disorder [22,26,29,30,31,32]. In this view, obsessions and compulsive behaviours take place in order to avoid situations that can trigger guilt, namely, to avoid the perception of being responsible for the occurrence of negative events [33]. These possible negative outcomes are perceived as essential to prevent. They may be actual, i.e., having consequences in the real world, and/or at a moral level [34]. Results from numerous studies using questionnaires [35] or experimental manipulations [36] suggest that obsessive symptoms are aimed at preventing, reducing, or neutralising the possibility of being guilty and responsible [37], sustaining evidence for such a link between responsibility and obsessive–compulsive symptoms. In OCD, an excessive engagement to deontological morality is present [28,38,39], which leads in turn to hyper-prudential checking behaviours. Control of thought processes and obsessive doubts are useful to avoid deontological guilt and a sense of responsibility [28]. In OC patients, the possibility of being guilty is evaluated by itself as a catastrophe, and it is represented as something unforgivable and unbearable [28,40]. On the other hand, the feeling of guilt can work as a signal of a possible looming threat (‘If I feel guilty, the event I have to prevent will occur’) [41]. According to the Diagnostic and Statistical Manual of Mental Disorders (DSM-5), it is possible to make a diagnosis of OCD even in children and, moreover, the early-onset of obsessive–compulsive disorder is one of the more common mental illnesses of children and adolescents, with a prevalence of 1% to 3% [42]. Furthermore, clinical manifestations of OCD in adolescents are very similar to those observed in adults [42,43]. 

On the opposite side of an imaginary morality continuum seems to be the conduct disorder (CD) diagnosis, which results in aggressive and antisocial behaviours. In fact, some authors consider it as the outcome of a total lack of morality. Recent studies have shown that guilt has the role of inhibiting antisocial behaviour [44,45,46,47] and plays a central role in predicting a high level of antisocial conduct. Diffusion of responsibility [48], a phenomenon in which people, when in a group, feel less responsible for the negative outcome of their behaviour compared to a situation in which they act alone, has been found to be the primary reason for increased antisocial and aggressive behaviours. Numerous studies have reported that, in people with problematic conduct, a fearless temperament, marked by behavioural disinhibition, prepares for a lack of moral reasoning that does not allow these patients to gain an internal system of rules [49,50]. These results seem to show that the total absence of guilt and empathy combine to predict very high levels of antisocial behaviours, proactive aggression, and school bullying [51,52]. As previously noted, empathy and guilt are considered moral emotions because they help to encourage prosocial behaviours [53]. Adolescents with CD are described as not feeling guilty [12]. Normally, developing children will increasingly internalise moral attributions and judgments [54]. However, this process of internalisation will be hindered if the negative arousal associated with guilt is attenuated due to the child’s temperament [12]. An opposite view about patients with a CD diagnosis posits that they feel guilty but consider anti-social purposes, such as dominance for example [55], as more important compared to moral purposes [56]. It could be plausible that these patients do have an internal system of moral rules but that they are considered at the same level as conventional ones [57]. Thus, the outcome of aggressive behaviours is a mechanism of moral disengagement [58] that acts by leading the deontological guilt to decrease. In line with this view, it might be assumed that patients with CD feel guilty, but this emotion is assessed as something from which to defend themselves. The lack of credit to morality also depends on the limited importance of moral goals compared to the high relevance of antisocial goals, including dominance, for example [55]. In this view, moral lack can occur not because of a patient’s lack of empathy or their fearless temperament, but because patients diagnosed with CD do not picture the moral consequences of the pain they can induce in others [56]. Namely, they may not anticipate the feeling of guilt or excess responsibility that would result from hurting others. Finally, clinical experience highlights how some beliefs in CD children (‘Authorities and their rules are unreliable and arbitrary’; ‘Nobody really cares about me’; ‘Since I have suffered, then the world is in credit to me’) induce the child to renounce affiliation with healthy peer groups [59] and to consider subordination to authority as useless and dangerous [60].

Therefore, in our opinion, although OCD and CD express a different symptomatology in adolescents, they could represent different answers to a common matrix about morality. In both diagnoses, an excessive sense of responsibility seems to be something that is unacceptable. In OC diagnosis, the patient feels guilty, and the sense of responsibility is hard to accept; the patient has to reassure themselves to not feel guilty and is not forgiving until a solution is found. In CD patients, the adolescent at first feels guilty and then tries to defend themselves by acting with anger to the authorities, who are considered unfair [61], by showing themselves to be someone who does not care about other people’s feelings, pain, and suffering.

Self-forgiveness and interpersonal forgiveness could represent a variable that determines the shift into a disorder or into the other. Our hypothesis is that this variable, i.e., the ability to forgive, might modulate different behavioural outcomes. 

Forgiveness has been shown to be an effective aspect in regulation of negative emotions [62,63,64]. It has been defined as ‘a willingness to abandon one’s right to resentment, negative judgement, and indifferent behaviour towards one who has unjustly hurt us’ [65]. When people forgive, there is a reduction in angry and resentful emotions, thoughts and behaviours, and an increase in positive and benevolent ones towards the offending person [66]. It has also been reported that promoting forgiveness increases anger control and reduces trait anger and anger expressions [67,68]. The forgiving inclination may act as a protective factor against the detrimental effects of dysfunctional behaviours triggered by anger. Most of the literature, in studying forgiveness, often refers to the willingness to forgive others when a wrongdoing has been done. In fact, although the recent literature has paid remarkable attention to the role of forgiveness in interpersonal relationships, less effort has been put into studying self-forgiveness. In the psychological literature, self-forgiveness has been defined as ‘a willingness to abandon self-resentment in the face of one’s own acknowledged objective wrong, while fostering compassion, generosity, and love toward oneself’ [65] (p. 115). Some research on the topic found that being unable to forgive oneself is associated with lower self-esteem and life satisfaction and higher neuroticism, depression, anxiety, and hostility [69,70,71,72]. In addition, Thompson and colleagues [69] introduced another dimension of forgiveness, namely, situation-forgiveness, defined as the willingness to forgive situations beyond anyone’s control (e.g., an illness or natural disaster).

Considering the abovementioned literature, in the present study performed on a non-clinical adolescent sample, the role of forgiveness in responsibility and guilt levels was investigated. We additionally tested whether this putative relation was influenced by tendencies towards obsessive–compulsive problems (OCP) or conduct problems (CP), i.e., whether forgiveness mediated the effect of OCD or CD symptoms on guilt and responsibility.

## 2. Materials and Methods

The study was conducted according to the guidelines of the Declaration of Helsinki and approved by the Ethics Committee of the Italian Association of Cognitive Psychotherapy (Associazione di Psicoterapia Cognitiva, APC) (protocol code: 3/2021; date of approval: 28 April 2021). Informed consent was obtained from all subjects involved in the study. For participants aged under 18 years, informed consent was signed by both parents. Participants did not receive any incentive for participating in the study. All research procedures conformed to the Declaration of Helsinki for Human Subjects or its later Amendments. 

### 2.1. Participants

Participants were 261 Italian students (52.3% F) attending year III, IV, or V of five different secondary schools in southern Italy. Participants were aged between 16 to 18 years (M = 17.37; SD = 0.84). Thirty subjects were excluded from the final sample due to incomplete tests. The final size of the sample was 231 subjects (110 M; 121 F). Subjects were naïve to the objectives and the predictions of the study.

### 2.2. Procedure

Participants were administered a booklet in the pencil and paper version, including all measures reported in the following ‘Questionnaire measures’ section. Administration of the questionnaires took place in the participants’ classroom during regular class hours under researcher supervision. Students were instructed to work alone and not to talk to each other while completing the questionnaires. The order of the presentation of the instruments was randomised.

### 2.3. Questionnaire Measures

#### 2.3.1. Personal Information Form

Participants first completed the folder in an anonymous way: they were asked to generate a personal code (given by the first letter of their mother’s name + their date of birth + exact number of their brothers and sisters) and to copy it on each sheet of the questionnaire set. Participants were then asked to indicate information about their gender, age, and school level. 

#### 2.3.2. Symptomatic Tendencies

The Youth Self-Report (YSR) [73,74] is a 112-item self-report questionnaire that evaluates adolescents’ behaviours during the recent 6 months by using a three-point Likert scale (from 0 never true to 2 sometimes true). Through the sum of different items, it is possible to calculate for each participant a total problem score, two summary scores (internalising and externalising problems), and syndrome scales based on DSM (Diagnostic and Statistical Manual, APA) syndromes. In the present study, the Obsessive–compulsive Scale (YSR-OCS) and Conduct Problems Scale (YSR-CPS) were used. 

#### 2.3.3. Forgiveness

The Heartland Forgiveness Scale (HFS) [65,75] is an 18-item self-report questionnaire that measures the person’s dispositional forgiveness (Total HFS), forgiveness of the self (Self-forgiveness), forgiveness of a particular situation (Situation-forgiveness), and forgiveness of the others (Others-forgiveness). 

#### 2.3.4. Responsibility

The Child Responsibility Attitude Scale (CRAS) is a self-report measure which has been adapted from the adult Responsibility Attitude Scale (RAS) [76]. The CRAS is a measure of general responsibility attitudes and consists of 20 items that ask the child to rate a series of statements on a seven-point Likert scale. Higher levels on this scale mean a lower responsibility attitude. Since the Italian version of the CRAS is not yet available, for the present study, a version independently translated by two professional interpreters was used. 

#### 2.3.5. Guilt

The Test of Self-Conscious Affect (TOSCA-A) [77] is a scenario-based measure of characteristic guilt-proneness and shame-proneness. The original scale consists of 15 scenarios, generated from adult participants’ descriptions of personal experiences of guilt, shame, and pride, followed by potential responses reflecting different affective tendencies. Respondents rate each response on a five-point Likert scale indicating how likely they would be to react in the manner described (1 = not likely, 5 = very likely). Ratings are summed across scenarios to obtain indices of guilt-proneness, shame-proneness, externalisation of blame, detachment/unconcern, alpha pride (i.e., pride in self), and beta pride (i.e., pride in behaviour). Since the Italian version of the TOSCA-A is not yet available, for the present study, a version independently translated by two professional interpreters was used.

### 2.4. Statistical Analyses

Data analyses were carried out using IBM SPSS, version 18. The α level was set at *p* < 0.05. For the assessment of normality, the Kolmogorov-Smirnov (K-S) test was used.

At first, to analyse the relationship between variables taken into account in the study, Pearson correlation coefficients were calculated. Then, a hierarchical regression analysis was conducted in which it was determined which variable, among three types of forgiveness (independent variables; IV), significantly predicted responsibility levels. Another hierarchical regression analysis was conducted to test the effect of the same IV on guilt levels. With the aim of testing a mediation model in which the putative effect of OCP or CP scales on responsibility levels was mediated by dimensions of forgiveness, two mediation analyses (one for each tendency scale) were performed using the bootstrap method. The last two mediational analyses were then conducted with the aim of investigating whether the two tendency scales (OCP or CP) influenced guilt levels and whether this effect was mediated by forgiveness. 

## 3. Results

Mean and standard deviations of all measures taken into account as part of the study are reported in Table 1. 

Correlational analysis revealed no significant relations among two problematic tendencies and age. CP levels were negatively related to guilt levels to a significant degree, and no significant correlations were found with three types of forgiveness and responsibility. Conversely, higher levels of OCP were found to be negatively related to self-forgiveness and situation-forgiveness and positively related to responsibility levels. No significant correlation was found between OCP and guilt. See Table 2 for the correlational analysis results.

The first regression analysis showed that age (B = −2.304; β = −0.134; t = −2.179; *p* = 0.030) and self-forgiveness (B = 0.938; β = 0.403; t = 6.704; *p* = 0.000) significantly predicted responsibility levels, explaining almost 20% of the total of variance (R^2^adj = 0.177; F_3227_ = 17.488; *p* = 0.000). The results revealed in detail that higher levels of self-forgiveness, along with younger age, predicted lower levels of perceived responsibility. The second regression analysis revealed that self-forgiveness (B = −0.347; β = −0.275; t = −3.509; *p* = 0.001) and situation-forgiveness (B = 0.214; β = 0.188; t = 2.229; *p* = 0.001) significantly predicted guilt levels. Specifically, lower levels of self-forgiveness and higher levels of situation-forgiveness predicted higher levels of guilt (R^2^adj = 0.067; F_3225_ = 14.315; *p* = 0.001). 

The mediation analyses were carried out using the PROCESS 3.1 macro for SPSS [78]. It employs a bootstrapping method for estimating indirect effects; 95% bias-corrected confidence intervals were calculated through 5000 bootstrap samples. From the variety of models proposed by the program, representative of different conceptual models, Model 4 was tested because it considers the effect of IV on the DV through mediators. Specifically, further mediational analyses were conducted that considered the regression results, namely, by not considering others-forgiveness in models in which the DV was guilt and not considering situation-forgiveness and others-forgiveness in models in which the DV was responsibility. 

**Figure 1:**
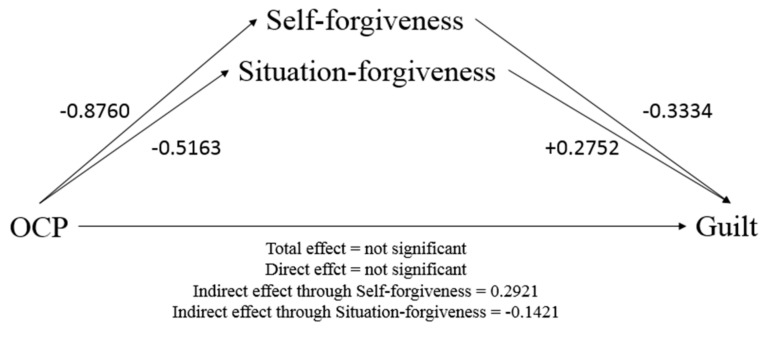
Mediation model tested: Obsessive–compulsive Problems (IV) on Guilt (DV) through Self-forgiveness and Situation-forgiveness (Mediators). Numbers reported are coefficient (B) of the effect represented. Coefficients are presented in Table 3.

Mediational analysis tested the model in which OCP was inserted as the IV and guilt as the DV; self-forgiveness and situation-forgiveness were settled as putative mediators, revealing that OCP does not exert significant direct and total effects on guilt levels, but rather has an indirect effect through the occurrence of both self-forgiveness and situation-forgiveness (Table 3). A suppression effect [79] was probably revealed: namely, a non-significant total effect of the IV on the DV due to the opposite in sign direct and indirect effects of the IV on the DV. Specifically, higher OCP led both self- and situation-forgiveness to decrease, which in turn led guilt levels to increase (self-forgiveness) and to decrease (situation-forgiveness). This resulted in a positive indirect effect exerted by self-forgiveness, by increasing guilt, and a negative indirect effect through situation-forgiveness, by decreasing guilt (Figure 1).

The same mediational analysis, with the same DV and putative mediators, was then carried out, replacing OCP with CP. The results showed no effects of CP on either self- or situation-forgiveness. Higher CP levels led guilt to decrease, but no indirect effects were revealed (Table 4). 

**Figure 2:**
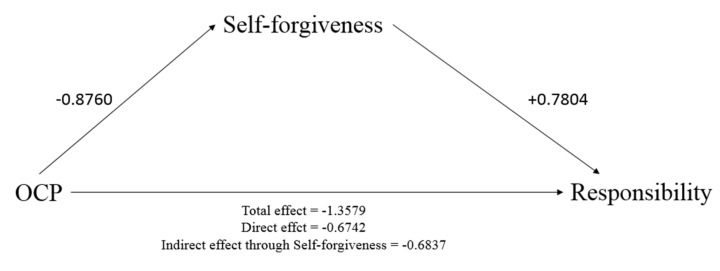
Mediation model tested: Obsessive–compulsive Problems (IV) on Responsibility (DV) through Self-forgiveness (Mediator). Numbers reported are coefficient (B) of the effect represented. Coefficients are presented in Table 5.

Two further mediational analyses were conducted to test mediation models in which the effect of OCP or CP on responsibility was mediated by self-forgiveness. The first analysis revealed that higher OCP led responsibility levels to increase both directly and indirectly through self-forgiveness. That is, higher OCP levels led self-forgiveness to decrease, which in turn led responsibility scores to decrease: the multiplication of these effects gave a positive indirect effect such that higher OCP levels led responsibility to increase through the mediation of self-forgiveness (Table 5 and Figure 2).

The second mediational analysis was carried out assuming that the effect of CP on responsibility levels was mediated by self-forgiveness, but this analysis actually showed no significant effects (either direct or indirect) of the IV on the DV (Table 6). 

## 4. Discussion

The aim of the present study was to investigate the role of forgiveness in the modulation of guilt and responsibility in a non-clinical sample of adolescents. Specifically, in the present study, the role of forgiveness was investigated in regard to responsibility and guilt levels, and we tested whether this putative relation was influenced by tendencies towards OCP or CP, i.e., whether specific dimensions of forgiveness mediate the effect of OCD or CD symptoms on guilt and responsibility.

Regarding the role of guilt in the two problematic trends (i.e., OCP and CP), our results confirmed the hypothesis that high levels of OCP are strongly related with high sensitivity to guilt [22,26,27,28], confirming that in OCD, the control of thought processes and obsessive doubt seem to be useful tools to avoid an ethical sense of guilt [28]. However, in relation to the absence of a correlation found between the OCP and TOSCA-A scores, we believe that this data may be due to the inability of the latter tool to grasp two different types of guilt, i.e., deontological and altruistic [56]. Our results also demonstrated that adolescents with a tendency towards CD actually feel guilty, in contrast to the position of Frick (2014), but less than adolescents without this problematic tendency, supporting alternative hypotheses for which they first feel guilty but develop processes that lead them to consider antisocial purposes as more important compared to moral purposes [56]; alternatively, they have an internal system of moral rules, but these rules are considered at the same level as conventional ones [57]. Regarding responsibility, our results confirmed only its role in the OCP tendency, whilst no effect was observed in CP. While these results support the idea that excessive responsibility is a central cognitive variable associated with OCP [22,26,29,30,31,32], they also suggest that, in adolescents with a tendency towards CP, no different levels of responsibility are observed compared to adolescents without this tendency. Taken together, our results regarding guilt and responsibility levels in adolescents with tendencies towards OCP or CP seem to confirm our hypothesis that although OCD and CD express a different symptomatology in adolescence, they could represent different answers to a common matrix about morality. Further research considering clinical samples are needed in order to better delineate these trajectories in diagnosed patients. 

Regarding the role of forgiveness in levels of responsibility and experienced guilt, our results revealed that self-forgiveness has an important role in modulating levels of a sense of responsibility, while either self-forgiveness or situation-forgiveness play a crucial role in guilt levels. In both regression analyses, the R^2^ values were not very high, with a very low coefficient in the regression that had guilt as the DV; this was probably because few putative predictors were inserted in the model. Indeed, guilt and responsibility are very complex constructs in which many variables (see, for example, social context and external models of emotion regulation) not considered in the present study have a modulating role. Therefore, further research considering a wider set of putative predictors is needed in order to improve our comprehension of the relationship between self-forgiveness, guilt, and responsibility. Moreover, mediation analyses revealed that two types of forgiveness (i.e., self- and situation-forgiveness) mediated the effect of OCP tendency on guilt levels. Furthermore, self-forgiveness mediated the role of OCP on responsibility levels. Regarding the effect of CP on guilt, this was not mediated by forgiveness. No effects of CP on responsibility levels were revealed. Thus, our results confirmed the need to include self-forgiveness in future models of forgiveness [72,80,81] for both theoretical and clinical implications. Moreover, the results of the present study highlight for the first time how the relation between self-forgiveness, guilt, and responsibility could lead to pathological outcomes, such as OCD. In fact, although our study was performed on a non-clinical sample, as Mancini and colleagues [43] stated, there is a ‘connection between normal and pathological obsessions and thus a similarity in the cognitive processes involved’, suggesting that non-clinical and clinical samples may differ more in degree than in kind [82]. In other words, in adolescents with higher levels of OCP, an increase in guilt and responsibility is considered to be something unacceptable and something to defend oneself against; these adolescents do not forgive themselves until they find a solution. The relationship between an inflated sense of responsibility and guilt and low proneness to self-forgiveness could be considered a vicious cycle underlying OCD. The present results seem to support its necessity in OCD: as stated by Barcaccia, Tenore, and Mancini [83], ’besides the already available treatments, it would be advantageous to design protocols aimed […] at developing a more general compassionate and forgiving stance towards oneself’ [84,85].

On the contrary, adolescents with higher CP levels experience guilt, albeit less than the rest of the sample and even if it is not affected by the degree of forgiveness. This result could be in line with a hypothesis in which CD patients at first feel guilty and then try to defend themselves by appearing to be someone who does not care about other people’s feelings, pain, and suffering.

Finally, forgiveness of the situation, namely, the willingness to forgive situations beyond anyone’s control (for example, an illness or a natural disaster), was also a significant predictor of the level of guilt in our results, confirming the importance of considering it in further conceptual models. More specifically, our results showed that adolescents who experienced higher levels of forgiveness of the situation experienced more guilt. This seems to represent a process whereby people who are faced with internal or external events (for example, an illness) are called upon to decide whether or not they have personal responsibility for that situation. When they attribute responsibility to themselves, then the situation (external or internal) is ‘discharged’. 

## 5. Limitations and Strengths

Although this study was the first to investigate an important issue, i.e., the role of forgiveness in guilt and responsibility in tendencies towards OCP and/or CP, some limitations need to be acknowledged. First of all, the results of the study are based on self-reported measures, which, especially in the clinical field, could represent a problem in terms of the generalisability of the results. Adolescents could, in fact, not be fully aware of how their thoughts or behaviours could represent a problematic issue in terms of pathology. The second limitation might be in not having considered clinical samples composed of OCP and CP diagnosed groups, which could have clarified the role of forgiveness in guilt and responsibility when the presence OCD and CD symptoms is problematic. Finally, the TOSCA-A was found to be a tool unable to grasp two different types of guilt, i.e., deontological and the altruistic. This probably affected the results regarding guilt. Further investigations should include instruments more able to make this very relevant distinction. 

## 6. Conclusions

In conclusion, the results of the present study show that a lower proneness to forgiveness increases responsibility and guilt and that this is particularly evident in subjects with higher levels of OCP. The present study is the first to highlight the important role of forgiveness in experiencing guilt and responsibility, particularly in adolescents with a higher presence of OC traits. Moreover, it initially evidenced the role of the interplay between these variables in a shift along the potential symptom spectrum (see OCP). 

## Figures and Tables

**Table 1:** Means and standard deviations of all measures taken into account as part of the study.

	Mean	Std. Deviation
YSR_OCS	5,33	3450
YSR_CPS	2,77	3113
HFS_TOT	82,43	15,503
Self-forgiveness	29,09	6208
Others-forgiveness	26,79	6520
Situation-forgiveness	26,65	6884
Responsibility (CRAS)	68,87	14,465
Guilty (TOSCA)	53,61	7846

Note. YSR_OCS = Obsessive–compulsive Scale; YSR-CPS = Conduct Problems Scale; HFS_TOT = Heartland Forgiveness Scale total score.

**Table 2:** Correlations among the study variables.

	1.	2.	3.	4.	5.	6.	7.
1. Self-forgiveness	-	0.208 **	0.571 **	−0.145 *	413 **	−0.487 **	−0.075
2. Others-forgiveness		-	0.419 **	0.158 *	0.017	0.029	−0.122
3. Situation-forgiveness			-	0.081	0.216 **	−0.259 **	−0.111
4. Guilt				-	−0.266 **	0.106	−0.185 **
5. Responsibility					-	−0.324 **	0.066
6. OCP						-	0.231
7. CP							-

Note. *p* < 0.05 *; *p* < 0.01 **.

**Table 3:** Results of the mediation analysis (Model 4) testing the effects of obsessive–compulsive problems (IV) on guilt (DV) through self-forgiveness and situation-forgiveness (mediators).

Model Summary	*R-Sq*	*F*	*p*	
	0.062	4.996	0.002	
***Model***	***B***	***t***	***p***	***CI***
Constant	55.493	16.602	<0.001	[48.906; 62.079]
Obsessive–compulsive Problems	−0.090	0.538	0.591	[−0.240; 0.420]
Self-forgiveness	−0.333	−3.046	0.003	[−0.549; −0.118]
Situation-forgiveness	0.275	3.082	0.002	[0.099; 0.451]
**Total Effects of IV on DV (*R-Sq* = 0.011; *F* = 2.579; *p* = 0.110)**				
	***Effect***	***t***	***p***	***CI***
Constant	52.327	55.186	<0.001	[50.459; 54.195]
Obsessive–compulsive Problems	0.240	1.606	0.110	[−0.055; 0.535]
**Significant Relative Indirect Effect of IV on DV Through Mediators**				
***Mediators***	***Effect***		***CI***	
Self-forgiveness	0.292		[0.095; 0.519]	
Situation-forgiveness	−0.142		[−0.271; −0.050]	

**Table 4:** Results of the mediation analysis (Model 4) testing the effects of conduct problems (IV) on guilt (DV) through self-forgiveness and situation-forgiveness (mediators).

Model Summary	*R-Sq*	*F*	*p*	
	0.093	7.778	<0.001	
***Model***	***B***	***t***	***p***	***CI***
Constant	58.612	22.765	<0.001	[53.539; 63.686]
Conduct Problems	−0.457	−2.851	0.004	[−0.773; −0.141]
Self-forgiveness	−0.363	−3.728	<0.001	[−0.556; −0.171]
Situation-forgiveness	0.256	2.904	0.004	[0.082; 0.429]
**Total Effects of IV on DV (*R-Sq* = 0.034; *F* = 8.072; *p* = 0.005):**				
	***Effect***	***t***	***p***	***CI***
Constant	54.897	80.510	<0.001	[53.553; 56.240]
Conduct Problems	−0.465	−2.841	0.005	[−0.788; −0.143]

**Table 5:** Results of the mediation analysis (Model 4) testing the effects of obsessive–compulsive problems (IV) on responsibility (DV) through self-forgiveness (mediator).

Model Summary	*R-Sq*	*F*	*p*	
	0.191	26.821	<0.001	
***Model***	***B***	***t***	***p***	***CI***
Constant	49.767	8.896	<0.001	[38.744; 60.790]
Obsessive–compulsive Problems	−0.674	−2.357	0.019	[−1.238; −0.111]
Self-forgiveness	0.780	4.910	<0.001	[0.467; 1.094]
**Total Effects of IV on DV (*R-Sq* = 0.105; *F* = 26.825; *p* = 0.000)**				
	***Effect***	***t***	***p***	***CI***
Constant	76.111	45.761	<0.001	[72.833; 79.388]
Obsessive–compulsive problems	−1.358	−5.180	<0.001	[−1.874; −0.841]
**Significant Relative Indirect Effect of IV on DV Through Mediator**				
***Mediator***	***Effect***		***CI***	
Self-forgiveness	−0.684		[−1.013; −0.386]	

**Table 6:** Results of the mediation analysis (Model 4) testing the effects Conduct problems (IV) on Responsibility (DV) through Self-forgiveness (mediator).

Model Summary	*R-Sq*	*F*	*p*	
	0.180	25.077	<0.001	
***Model***	***B***	***t***	***p***	***CI***
Constant	39.102	9.107	<0.001	[30.642; 47.563]
Conduct Problems	0.456	1.632	0.104	[−0.095; 1.007]
Self-forgiveness	0.980	6.995	<0.001	[0.704; 1.256]
**Total Effects of IV on DV (*R-Sq* = 0.004; *F* = 1.014; *p* = 0.315)**				
	***Effect***	***t***	***p***	***CI***
Constant	68.018	*53.295*	<0.001	[65.504; 70.533]
Conduct Problems	0.309	1.007	0.315	[−0.295; 0.912]
**Significant Relative Indirect Effect of IV on DV Through Mediator**				
***Mediator***	***Effect***	***CI***		
Self-forgiveness	−0.148	[−0.399; 0.098]		

## Data Availability

The data presented in this study are available upon request of the corresponding author. The data is not publicly available because it was collected on a sample of minors.
